# Plasmofluidic Disk Resonators

**DOI:** 10.1038/srep23149

**Published:** 2016-03-16

**Authors:** Min-Suk Kwon, Bonwoo Ku, Yonghan Kim

**Affiliations:** 1 School of Electrical and Computer Engineering, Ulsan National Institute of Science and Technology, UNIST-gil 50, Ulsan 689-798, Republic of Korea

## Abstract

Waveguide-coupled silicon ring or disk resonators have been used for optical signal processing and sensing. Large-scale integration of optical devices demands continuous reduction in their footprints, and ultimately they need to be replaced by silicon-based plasmonic resonators. However, few waveguide-coupled silicon-based plasmonic resonators have been realized until now. Moreover, fluid cannot interact effectively with them since their resonance modes are strongly confined in solid regions. To solve this problem, this paper reports realized plasmofluidic disk resonators (PDRs). The PDR consists of a submicrometer radius silicon disk and metal laterally surrounding the disk with a 30-nm-wide channel in between. The channel is filled with fluid, and the resonance mode of the PDR is strongly confined in the fluid. The PDR coupled to a metal-insulator-silicon-insulator-metal waveguide is implemented by using standard complementary metal oxide semiconductor technology. If the refractive index of the fluid increases by 0.141, the transmission spectrum of the waveguide coupled to the PDR of radius 0.9 μm red-shifts by 30 nm. The PDR can be used as a refractive index sensor requiring a very small amount of analyte. Plus, the PDR filled with liquid crystal may be an ultracompact intensity modulator which is effectively controlled by small driving voltage.

Silicon photonic resonators are usually integrated-optical rings or disks in which light circulates to be stored at specific wavelengths. They are playing key roles in silicon photonics as modulators, switches, and filters^1^,^2^,^3^. In addition, they have attracted a lot of attention as compact sensors^4^,^5^,^6^. Nowadays, there is a strong demand that the footprints of silicon photonic resonators need to be reduced in order to increase the integration density of a silicon photonic integrated-circuit. Small ring or disk resonators have additional advantages such as a large free spectral range and use of a small amount of analyte in sensing application. Moreover, it is possible to achieve a large value of the Purcell factor *F*_*p*_, which leads to development of novel devices such as a single photon source^7^.

To satisfy the demand, there have been many researches on small silicon photonic resonators^8^,^9^,^10^. However, it is well known that silicon photonic resonators cannot be made small below the diffraction limit. Therefore, ultimate reduction of the effective mode volume (*V*_eff_) of a resonator may be obtained by switching from silicon photonic resonators to plasmonic resonators which support resonance modes with the nature of a surface plasmon polariton. A variety of individually addressable plasmonic resonators have been theoretically studied^11^,^12^,^13^,^14^,^15^, but just a few of them, especially silicon-based hybrid plasmonic ring or disk resonators, have been realized and characterized^16^,^17^,^18^. Silicon-based hybrid plasmonic ring or disk resonators support resonance modes which have electric field highly enhanced in the narrow region, with a low refractive index, between metal and silicon. They are prominent since they possess moderate values of the Q factor and small values of *V*_eff_. In addition, they are compatible with standard complementary metal oxide semiconductor (CMOS) technology such that seamless integration between them and silicon photonic devices is easy.

In this paper, plasmofluidic disk resonators (PDRs), which are silicon-based hybrid plasmonic disk resonators, are investigated. The PDR has a silicon disk laterally surrounded by metal with a narrow channel in between. The narrow channel is filled with fluid, and the resonance modes of the PDR are substantially confined in the fluid. The key feature of the PDR is that replacement of one fluid with another can reconfigure its resonance characteristics. This can be exploited to implement microfluidically controllable plasmonic devices and sensors requiring an extremely small volume of analyte, to name a few. This paper, first, explains the calculated characteristics of the isolated PDRs. Then, the PDRs side-coupled to metal-insulator-silicon-insulator-metal (MISIM) waveguides^19^,^20^,^21^,^22^ are fabricated by using standard CMOS technology and characterized. Their measured characteristics are compared with calculated ones. Finally, possible improvement and application of the PDR coupled to the MISIM waveguide are dicussed.

## Results

### Theoretical analysis of the isolated PDR

**Figure 1:**
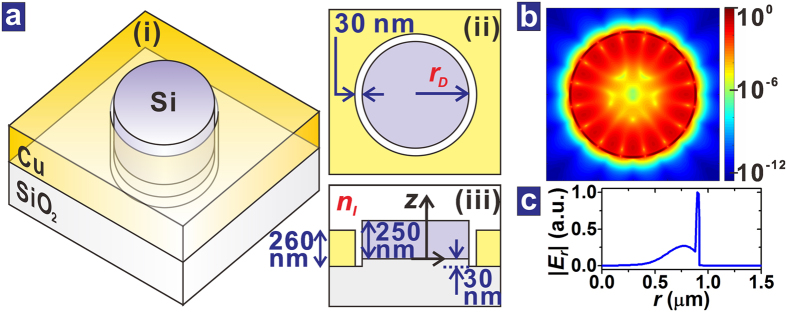
Isolated PDR. (**a**) Three-dimensional structure (i), top view (ii), and cross-sectional structure (iii) of the PDR. (**b**) Distribution of |**E**|^2^ of a PDR resonance mode at *z* = 125 nm on the log scale. (**c**) Distribution of |*E*_*r*_| of the PDR resonance mode. The PDR resonance mode is for *r*_*D*_ = 0.9 μm and *n*_*l*_ = 1.45 and it is centered at *λ* = 1558 nm.

The isolated PDR is schematically shown in Fig. 1(a). The silicon (Si) disk is 250 nm thick, and its radius is *r*_*D*_. The metal of the resonator is copper (Cu) of thickness 260 nm, and it is recessed 30 nm below the bottom of the Si disk. The circular channel between the Si disk and the metal is 30 nm wide. The PDR is covered by fluid of refractive index *n*_*l*_. The parameter values used for the isolated PDR come from those of the realized PDR coupled to the MISIM waveguide. The PDR was analyzed by using the finite difference time domain (FDTD) method (See Methods). *r*_*D*_ was appropriately adjusted such that a resonance mode exists at the free-space wavelength *λ* around 1550 nm. The resonance mode has a field distribution in which the major component of its electric field **E** is in the radial direction. For *r*_*D*_ = 0.9 μm and *n*_*l*_ = 1.45, the distribution of |**E**|^2^ at *λ* = 1558 nm in the plane at *z* = 125 nm is shown in Fig. 1(b). The electric field is highly enhanced in the channel. This is also confirmed from the distribution of the radial component of the electric field, *E*_*r*_ as shown in Fig. 1(c). Half the number of nodes in the azimuthal direction in Fig. 1(b) corresponds to the azimuthal order of the resonance mode, *m*. When *r*_*D*_ ≤ 1.4 μm, all the resonance modes in the wavelength range between 1400 and 1700 nm have similar field distributions in the azimuthal plane, i.e. there is no resonance mode of higher radial-order or higher vertical-order.

**Figure 2:**
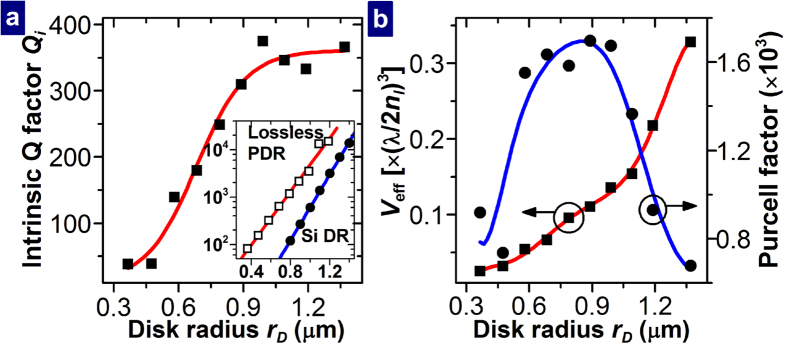
Calculated properties of the isolated PDR filled with fluid with *n*_*l*_ = 1.45. (**a**) Relation of the intrinsic Q factor of the PDR to the disk radius. The inset shows the Q factor of the lossless PDR, which corresponds to the radiation-limited Q factor of the PDR, and that of the Si disk resonator (DR) embedded in the fluid as functions of the disk radius. (**b**) Relations of the normalized effective mode volume and Purcell factor of the PDR to the disk radius. In all the figures, the symbols represent the values obtained from the FDTD simulation. The lines are guides for eyes. Especially, the line related to the Purcell factor was calculated from the lines related to *Q*_*i*_ and *V*_eff_.

The calculated relation between the intrinsic Q factor of the PDR, *Q*_*i*_ and *r*_*D*_ for *n*_*l*_ = 1.45 is shown in Fig. 2(a). If *r*_*D*_ ≤ 0.89 μm, *Q*_*i*_ increases with *r*_*D*_. For *r*_*D*_ > 0.89 μm, *Q*_*i*_ seems to saturate at ~350. *Q*_*i*_ satisfies the following relation: 1/*Q*_*i*_ = 1/*Q*_*r*_+1/*Q*_*o*_, where *Q*_*r*_ and *Q*_*o*_ are the Q factors determined by the radiation and Ohmic losses of the PDR. *Q*_*r*_ is obtained by analyzing the PDR with the imaginary part of the dielectric constant of Cu set to 0. As shown in the inset of Fig. 2(a), *Q*_*r*_ increases almost exponentially with *r*_*D*_. As *r*_*D*_ decreases, *Q*_*o*_ decreases since the field distribution in the PDR is further shifted outwards, especially toward the metal, for a smaller value of *r*_*D*_ such that the Ohmic loss due to the absorption by the metal increases. For *r*_*D*_ > 0.89 μm, *Q*_*i*_ is limited by the Ohmic loss, and *Q*_*i*_ ≈ *Q*_*o*_. In the case of a curved MISIM waveguide, its propagation loss was shown to approach that of a straight MISIM waveguide as the radius of curvature of the bend increases^22^. Similarly, the attenuation coefficient *α*_*D*_ of the resonance mode in the azimuthal direction approaches some value such that the saturation of *Q*_*i*_ which is inversely proportional to *α*_*D*_ exists. For comparison, a 250-nm-thick Si disk embedded in the fluid of refractive index *n*_*l*_ was also analyzed, and the dependence of its intrinsic Q factor on *r*_*D*_ is also shown in Fig. 2(a). For *r*_*D*_ < 0.92 μm, the PDR possesses a larger intrinsic Q factor than the Si disk resonator. The effective mode volume *V*_eff_ and Purcell factor *F*_*p*_ of the resonance mode were calculated (See Methods). The calculated values of *V*_eff_ and *F*_*p*_ are shown with respect to *r*_*D*_ in Fig. 2(b). Both the intrinsic Q factor and effective mode volume of the PDR are smaller than those of previous Si-based hybrid plasmonic disk resonators^11^,^18^, which are vertical stacks of metal, oxide, and Si disks. In consequence, the Purcell factor of the PDR is similar to those of the previous Si-based hybrid plasmonic disk resonators.

### Investigation of the PDR coupled to the MISIM waveguide

**Figure 3:**
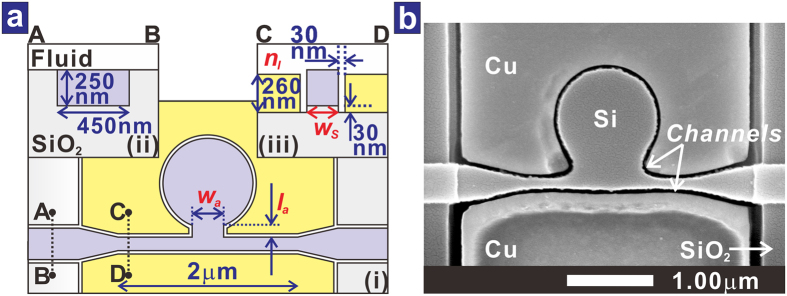
PDR side-coupled to the MISIM waveguide. (**a**) Schematic diagram of the PDR side-coupled to the MISIM waveguide in (i). The cross-sectional structures of the Si photonic waveguide and the MISIM waveguide are shown in (ii) and (iii), respectively. (**b**) Surface SEM image of the realized PDR side-coupled to the MISIM waveguide.

The PDR side-coupled to the MISIM waveguide is schematically shown in Fig. 3(a). The MISIM waveguide consists of the 250-nm-high Si strip of width *w*_*S*_ and the 260-nm-thick Cu patterns sandwiching the Si strip with the 30-nm-wide channels in between. These channels are also filled with the fluid, and the fluid is the insulator of the MISIM waveguide. There is a sort of aperture-assisted coupling^23^ between the PDR and the MISIM waveguide. The Si pattern of the aperture has a width *w*_*a*_ and a length *l*_*a*_. The MISIM waveguide is coupled to Si photonic waveguides with a 450-nm-wide Si core via 600-nm-long tapering regions. Each Si photonic waveguide is coupled to another silicon photonic waveguide with a 5-μm-wide Si core via a 200-μm-long tapering region. A scanning electron microscope image of the fabricated device is shown in Fig. 3(b). The length of the MISIM waveguide is 2 μm, and *w*_*S*_, *w*_*a*_, and *l*_*a*_ are about 190 nm, 1.0 μm, and 0.23 μm, respectively.

**Figure 4:**
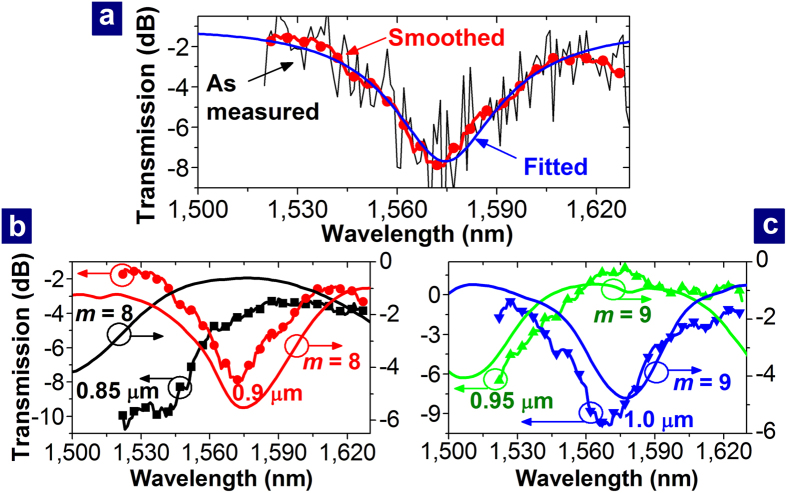
Transmission spectra of the PDR-coupled MISIM waveguides for different values of *r*_*D*_. (**a**) As-measured, smoothed, and fitted transmission spectra related to the PDR with *r*_*D*_ = 0.9 μm and *n*_*l*_ = 1.44. (**b,c**) Measured (lines with symbols) and Calculated (lines) transmission spectra for *r*_*D*_ = 0.85, 0.9, 0.95, and 1.0 μm.

The channels were filled with an index oil with *n*_*l*_ = 1.44. The as-measured transmission spectrum of the MISIM waveguide coupled to the PDR with *r*_*D*_ = 0.9 μm is shown in Fig. 4(a). Because of the Fabry-Perot resonance between the facets of the input and output 5-μm-wide Si waveguides, which are about 15 mm apart, there are rapid fluctuations. To remove the influence of the Fabry-Perot resonance, the as-measured transmission spectrum was smoothed by using the adjacent averaging method (See Supplementary Information S1), and the smoothed transmission spectrum is also shown in Fig. 4(a). Then, the theoretical spectrum of a simple resonator-coupled waveguide, which is expressed by


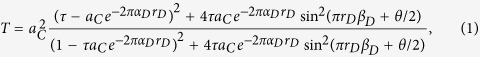


was fitted to the smoothed transmission spectrum (See Supplementary Information S2). In Eq. (1), *β*_*D*_ and *α*_*D*_ are the propagation constant and attenuation coefficient of the resonance mode in the azimuthal direction; *a*_*C*_ is related to the fractional loss of the coupling between the PDR and the MISIM waveguide, which is 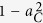; *τ* exp(i*θ*) is the straight-through coupling coefficient of the coupling. The fitted curve is also shown in Fig. 4(a). The loaded Q factor of the PDR is 24.6, and the value of *Q*_*i*_ of the realized PDR, which is determined from the fitting process, is 57 (See Methods). According to Eq. (1), the PDR is critically coupled to the MISIM waveguide (i.e., *T* = 0) if *τ* = *a*_*C*_ exp(−2*πα*_*D*_*r*_*D*_). The fitted values of *τ*, *a*_*C*_, and *α*_*D*_ are 0.7888, 0.9299, and 9.908 × 10^−2^ μm^−1^, respectively, and *τ* > *a*_*C*_ exp(−2*πα*_*D*_*r*_*D*_) for these values. Therefore, the PDR seems to be under-coupled to the MISIM waveguide. The value of *Q*_*i*_ is about 5.6 times smaller than that of the ideal PDR, which is shown in Fig. 2(a). The etching of silicon dioxide (SiO_2_) in the fabrication process (See Methods) seems to induce roughness on the rim of the Si disk. Because of this roughness, the attenuation coefficient *α*_*D*_ of the resonance mode in the azimuthal direction increases, and *Q*_*i*_ decreases. The decrease of *Q*_*i*_ accords with the increase of the propagation loss of the MISIM waveguide. The propagation loss is 0.30 dB/μm prior to the etching of SiO_2_ and it increases to 0.46 dB/μm after the etching and filling the channels with the fluid^24^. The measured transmission spectra of the MISIM waveguides coupled to the PDRs with different values of *r*_*D*_ are shown in Fig. 4(b,c) and compared with those calculated by using the FDTD method. As deduced from the resonance condition of the PDR, the transmission spectrum shifts toward a longer wavelength for a larger value of *r*_*D*_. The calculation results reveal that the transmission spectra in Fig. 4(b) are associated with the resonance mode of order 8 and those in Fig. 4(c) are associated with the resonance mode of order 9.

**Figure 5:**
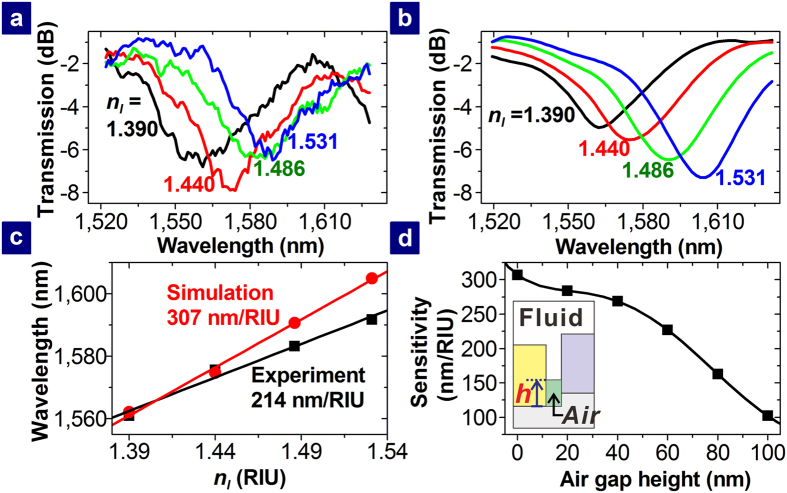
Transmission spectra of the PDR-coupled MISIM waveguide for different values of *n*_*l*_. (**a**) Measured and (**b**) calculated transmission spectra of the MISIM waveguide coupled to the PDR with *r*_*D*_ = 0.9 μm for different values of *n*_*l*_. (**c**) Measured and calculated relations (symbols) between the resonance wavelength and *n*_*l*_. The straight lines are fitted to the relations. (**d**) Relation of the sensitivity to the air gap height depicted in the inset. The symbols represent the values obtained from the FDTD simulation. The line is a guide for eyes.

The transmission spectrum of the MISIM waveguide coupled to the PDR with *r*_*D*_ = 0.9 μm was measured while the channels were filled with different index oils. The measured transmission spectra are shown in Fig. 5(a) for different values of *n*_*l*_. As *n*_*l*_ increases, the transmission spectrum further red-shifts. The shift of the transmission spectrum depending on *n*_*l*_ is in good agreement with the calculated one shown in Fig. 5(b). The measured and calculated relations of the resonance wavelength *λ*_*m*_ (i.e., the center wavelength of the transmission spectrum) to *n*_*l*_ are shown in Fig. 5(c). The slope of the straight line fitted to the measured relation is 214 nm/RIU (RIU is the acronym of refractive index unit), which is about 70% of the calculated slope. The difference between the measured and calculated slopes may be caused by the partially filled channels. To check this, the PDR-coupled MISIM waveguide with air gaps of height *h* at the bottom of the channels (See the inset of Fig. 5(d)) was simulated for a few values of *h* and *n*_*l*_ = 1.390, 1.440, 1.486, and 1.531. For a fixed value of *n*_*l*_, *λ*_*m*_ decreases and the minimum transmission at *λ*_*m*_ increases as *h* increases (See Supplementary Information S3). In addition, such changes become more considerable for a larger value of *n*_*l*_. This happens since the coupling between the MISIM waveguide and the PDR is affected by the air gaps. From the relation of *λ*_*m*_ to *n*_*l*_, the sensitivity for a value of *h* was calculated. The relation of the sensitivity to *h* is shown in Fig. 5(d). As *h* increases, the sensitivity decreases. The value of *h* at which the sensitivity is the same as the experimental value is estimated to be 64 nm. The air gaps may exist since a drop of the index oil was applied to the surface of the PDR-coupled MISIM waveguide and the index oil filled the channels downwards from the surface. If the channels are sealed by bonding appropriate polymer like poly(methyl methacrylate) (PMMA)^25^, they may be filled by capillary flow^26^ or electrokinetic flow^27^,^28^,^29^ without the air gaps.

If the PDR coupled to the MISIM waveguide is considered as a refractive index sensor, the slope corresponds to the sensitivity of the sensor. The sensitivity of the PDR is more than two times larger than those of Si disk resonators^6^. However, the measures of evaluating a refractive index sensor are not only the sensitivity but also the linewidth of the transmission spectrum of the sensor. The figure of merit for refractive index sensors is the ratio of the sensitivity to the linewidth. The figure of merit of a large Si disk resonator is much larger than that of the PDR. However, *Q*_*i*_ of a Si disk resonator becomes smaller than *Q*_*i*_ of the PDR when *r*_*D*_ is smaller than 0.92 μm as shown in Fig. 2(a). This means that the linewidth, which is inversely proportional to *Q*_*i*_, of the PDR becomes smaller than that of a Si disk resonator. Therefore, in a situation that requires a compact refractive index sensor in need of a small amount of analyte, the figure of merit of the PDR becomes larger than that of a Si disk resonator if *r*_*D*_ ≤ 0.92 μm. Although the figure of merit of the realized PDR is about 3.3, it may reach up to 9.7 if the fabrication-induced loss is reduced to zero and the PDR is critically coupled to the MISIM waveguide as discussed below. Despite the modest figure of merit, the PDR is attractive as a sensor since it requires an extremely small volume of analyte, which ultimately approaches the volume of the channel. The volume of the channel is estimated to be 44.8 attoliters if the channel is completely filled without an air gap.

### Improvement and application of the PDR coupled to the MISIM waveguide

**Figure 6:**
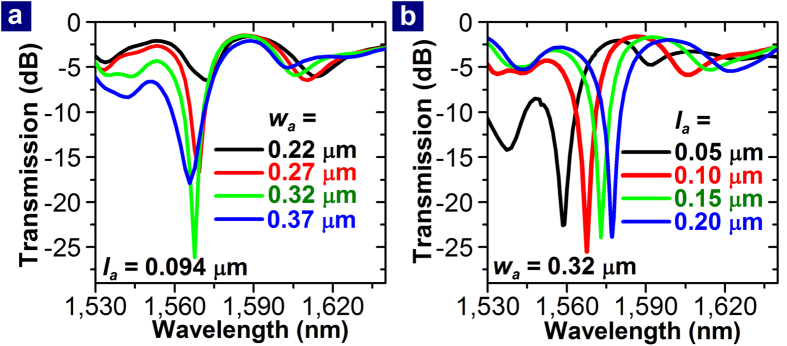
Dependence of the transmission spectrum on *w*_*a*_ and *l*_*a*_. The transmission spectra of the MISIM waveguide with *w*_*S*_ = 50 nm which is coupled to the PDR with *r*_*D*_ = 0.9 μm are shown for various values of (**a**) *w*_*a*_ and (**b**) *l*_*a*_.

The PDR-coupled MISIM waveguide needs to be improved such that the resonance band in its transmission spectrum becomes deeper and narrower. For this purpose, the aperture-assisted coupling between the PDR and the MISIM waveguide needs to be controlled by adjusting the width (*w*_*a*_) and length (*l*_*a*_) of the aperture region. FDTD simulation shows that *w*_*a*_ more influences on the coupling than *l*_*a*_(See Supplementary Information S4). However, when *w*_*S*_ = 190 nm, the PDR with *r*_*D*_ = 0.9 μm cannot be critically coupled to the MISIM waveguide regardless of *w*_*a*_. If *w*_*S*_ is reduced to 50 nm, the PDR is almost critically coupled to the MISIM waveguide as checked from Fig. 6, which shows the transmission spectra of the PDR-coupled MISIM waveguide with *w*_*S*_ = 50 nm for various values of *w*_*a*_ and *l*_*a*_. For *w*_*a*_ = 320 nm and *l*_*a*_ = 100 nm, the minimum transmission at the resonance wavelength is −25.5 dB, the linewidth of the transmission spectrum is 22 nm, and the loaded Q factor of the PDR is 71.2.

The PDR or the PDR-coupled MISIM waveguide belongs to plasmofluidics which is emerging as an attractive field with synergetic advantages of plasmonics and micro- or nano-fluidics. Recently, there have been two review papers focusing on plasmofluidics^30^,^31^. Diverse applications of plasmofluidics are explained there, and one example of the applications is plasmonic sensing^32^ such as the aforementioned refractive index sensor based on the PDR. Another application is reconfiguration of plasmonic devices by means of liquid crystal (LC)^31^. With regard to this point, the PDR-coupled MISIM waveguide can be used as an ultracompact intensity modulator based on LC. When well-known LC E7 is used as fluid, it is anticipated that the transmission of the PDR-coupled MISIM waveguide can be tuned by about 17 dB with a driving signal of a few volts applied across the channel surfaces (See Supplementary Information S5).

## Conclusion

The PDR, which is an individually addressable Si-based hybrid plasmonic disk resonator, has been theoretically and experimentally investigated. For the realization of the PDR coupled to the MISIM waveguide, standard CMOS technology has been used. The measured characteristics of the PDR coupled to the MISIM waveguide have been compared with the characteristics calculated by using the FDTD method. It has been shown that the former are in good agreement with the latter. When the radius of the Si disk is 0.9 μm, the loaded Q factor of the PDR is 24.6, and its intrinsic Q factor is estimated to be 57. If the fabrication process is improved, the intrinsic Q factor is expected to increase up to the theoretical value of 317. The loaded Q factor is also likely to approach 71.2 if the structural parameters of the coupling region are appropriately adjusted and the width of the Si strip of the MISIM waveguide is reduced to 50 nm. As a refractive index sensor, the realized PDR coupled to the MISIM waveguide has a sensitivity of 214 nm/RIU and it is very attractive in terms of requiring an extremely small volume of analyte. The PDRs are expected to open the door to ultracompact devices exploiting functional fluids such as liquid crystal.

## Methods

### Fabrication

To realize the PDR coupled to the MISIM waveguide, first, this device with the channels filled with SiO_2_ was fabricated by using standard CMOS technology. The first step of the fabrication was low pressure chemical vapor deposition (LPCVD) of 50-nm-thick Si oxide and 100-nm-thick Si nitride, which was used as an etch stop for the following chemical mechanical polishing (CMP), on an 8-inch silicon-on-insulator wafer with 2-μm-thick buried oxide. Then, 248 nm optical lithography and dry etching were carried out to make Si patterns. Over the patterns, 800-nm-thick Si oxide was formed by using high-density plasma chemical vapor deposition. To make the Si oxide surface planar, CMP was done. For the following Cu damascene process, another 248 nm optical lithography and dry etching were carried out to selectively remove the Si oxide and expose the Si patterns associated with the MISIM waveguides and the PDRs. Then, a thin Si oxide layer was conformally covered over the exposed Si patterns by using LPCVD. Finally, 800-nm-thick Cu was deposited by sputtering, and Cu CMP was done until the top surface of the thin Si oxide layer was exposed. These steps are the same as in ref. 20 and they were carried out by using the fabrication services provided by National Nanofab Center (www.nnfc.re.kr). Identical chips with the PDR-coupled MISIM waveguides, which have dimensions of 20 mm × 15 mm, were made on the wafer. One chip was diced out of the wafer, and a SU-8 microfluidic channel was formed on it by patterning SU-8 (MicroChem, SU-8 2015). Then, the channel-filling SiO_2_ was etched away with the SU-8 pattern used as an etching mask. The chip was immersed into buffered oxide etchant solution (JT Baker, Model 1178-03) for 55 seconds, thoroughly rinsed with deionized water, and dried. Finally, the chip was diced to make the input and output facets of the Si photonic waveguides with a 5-μm-wide core. During the dicing, the SU-8 pattern again plays the important role of reducing dicing-induced damage to the Si photonic waveguides. These steps are the same as in ref. 24.

### Measurement

Two lensed fibers (of spot diameter 2.5 ± 0.3 μm, OZ Optics) was end-fire coupled to the 5-μm-core Si waveguides. Horizontally polarized light from a tunable laser (New Focus, Model TLB-6600) was launched into the input lensed fiber, and the light from the output lensed fiber was measured by an optical power meter (Newport, Model 2936-R). The transmission spectrum of the MISIM waveguide coupled to the PDR was normalized to that of the MISIM waveguide uncoupled to the resonator. Before the measurement, one of the index oils (Cargille, Series A, AA, and AAA) was dropped into the SU-8 channel and filled it. After the measurement, the chip was rinsed with methanol and deionized water, dried, and then reused.

### Simulation

The PDR was analyzed by using the FDTD method (FDTD Solutions, Lumerical Inc.). The dielectric constant of Cu was calculated using the material model built in FDTD Solutions, which is based on the measured dielectric constant in ref. 33. When the isolated PDR was analyzed, an electric dipole source aligned radially was placed inside the channel. When the PDR-coupled MISIM waveguide was analyzed, the fundamental mode of the MISIM waveguide, which is called a hybrid plasmonic mode^19^, was launched into the left side of the MISIM waveguide before the PDR. The power of the right side of the MISIM waveguide after the PDR was monitored. Similar to the measurement, the transmission which is the ratio of the monitored power to the launched power was normalized to that of the isolated MISIM waveguide with the same length as the PDR-coupled MISIM waveguide.

The effective mode volume *V*_eff_ of the resonance mode was calculated using the expression^34^


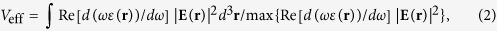


where **E**(**r**) is the electric field of the resonance mode and *ε*(**r**) is the dielectric constant distribution in the isolated PDR. In addition, the Purcell factor *F*_*p*_ was calculated by using 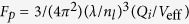.

For the fitting process, first, the isolated PDR with *r*_*D*_ = 0.9 μm was analyzed by using the bend mode solver of FIMMWAVE (Photon Design) based on the finite element method. This analysis provided the effective index *N*_*D*_ (=*β*_*D*_/(2*π*/*λ*_*m*_)) and group index *N*_*g*_ of the resonance mode. Then, the values of *a*_*C*_, *α*_*D*_, *τ*, and *θ* were determined from the fitting process. The intrinsic Q factor of the isolated PDR was calculated by using the relation *Q*_*i*_ = *πN*_*g*_/(*α*_*D*_*λ*_*m*_), and the loaded Q factor of the PDR coupled to the MISIM waveguide was given by the ratio of *λ*_*m*_ to the linewidth of the fitted transmission spectrum.

## Additional Information

**How to cite this article**: Kwon, M.-S. *et al*. Plasmofluidic Disk Resonators. *Sci. Rep*. **6**, 23149; doi: 10.1038/srep23149 (2016).

## Supplementary Material

Supplementary Information
