# Supplementary material for: Plasmofluidic Disk Resonators
Source: Sci Rep. 2016 Mar 16;6:23149. doi: 10.1038/srep23149 (PMC4793221; doi:10.1038/srep23149)
Supplement: Supplementary Information [file srep23149-s1.pdf]

Supplementary Information

Plasmofluidic disk resonators

Min-Suk Kwon, Bon Woo Ku, and Yonghan Kim*

School of Electrical and Computer Engineering, Ulsan National Institute of Science and Technology, UNIST-gil 50, Ulsan 689-798, Republic of Korea

* Corresponding Author: mskwon@unist.ac.kr

S1. As-measured and smoothed transmission spectra

As shown in Fig. 4(a), the as-measured transmission spectrum of the MISIM waveguide coupled to the PDR rapidly fluctuates. This rapid fluctuation is partly caused by the Fabry-Perot resonance of the chip. Since the transmission of only the MISIM waveguide coupled to the PDR slowly changes with the wavelength, the rapid fluctuation needs to be removed. For this purpose, the adjacent averaging method was employed. Let $T_A(\lambda_i)$ be the as-measured transmission at λ_i and $T_S(\lambda_i)$ be the transmission obtained by using the method. Then,

$$T_S(\lambda_i) = \frac{1}{N} \sum_{j=-(N-1)/2}^{(N-1)/2} T_A(\lambda_{i+j}), \tag{S1}$$

where N is an odd integer. Figure S1 shows how the smoothed transmission spectrum of the MISIM waveguide coupled to the PDR with r_D = 0.9 μm and n_l = 1.44 changes depending on N. If N is smaller than 7, the fluctuation still exists. If N is larger than 9, the smoothed transmission spectrum does not change significantly. From this result, N was set to 11. Figure S2 shows the as-measured and smoothed transmission spectra for r_D = 0.85, 0.9, 0.95, and 1.0

μm when n_l = 1.44. Figure S3 shows the as-measured and smoothed transmission spectra for n_l = 1.390, 1.440, 1.486, and 1.531 when r_D = 0.9 μm.

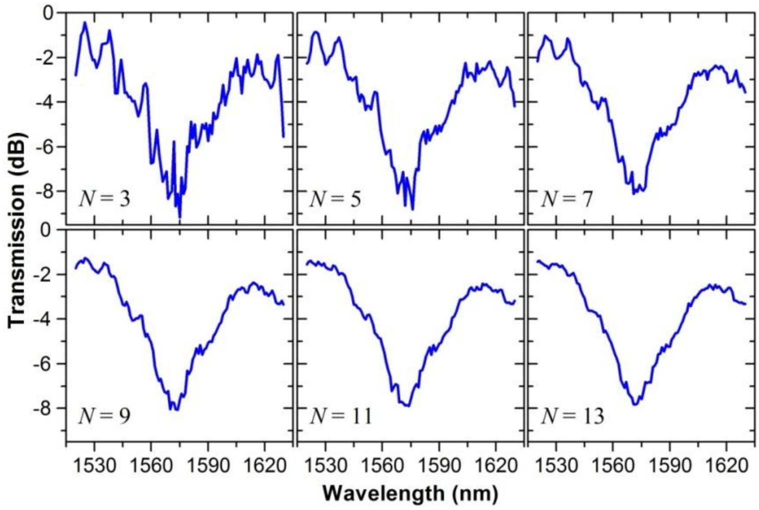

Figure S1. Smoothed transmission spectra of the MISIM waveguide coupled to the PDR with r_D = 0.9 μm and n_l = 1.44 for different values of *N*.

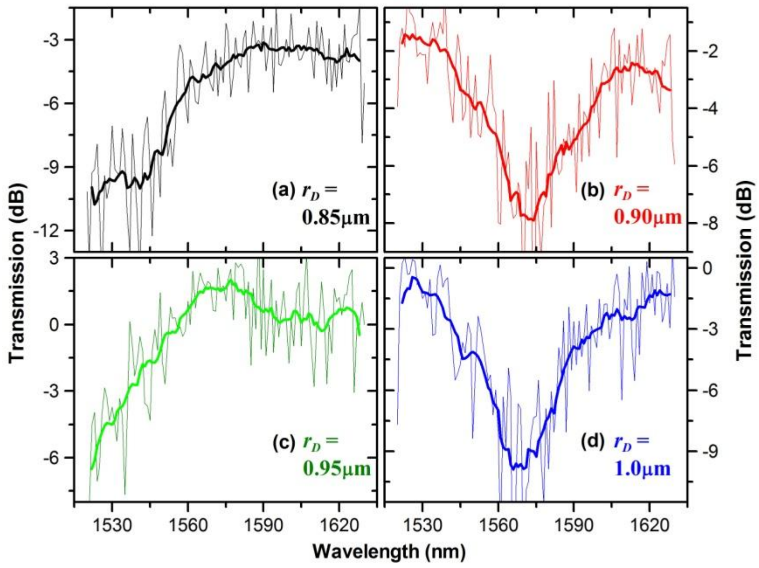

Figure S2. As-measured (thin lines) and smoothed (thick lines) transmission spectra for r_D = 0.85, 0.9, 0.95, and 1.0 μm

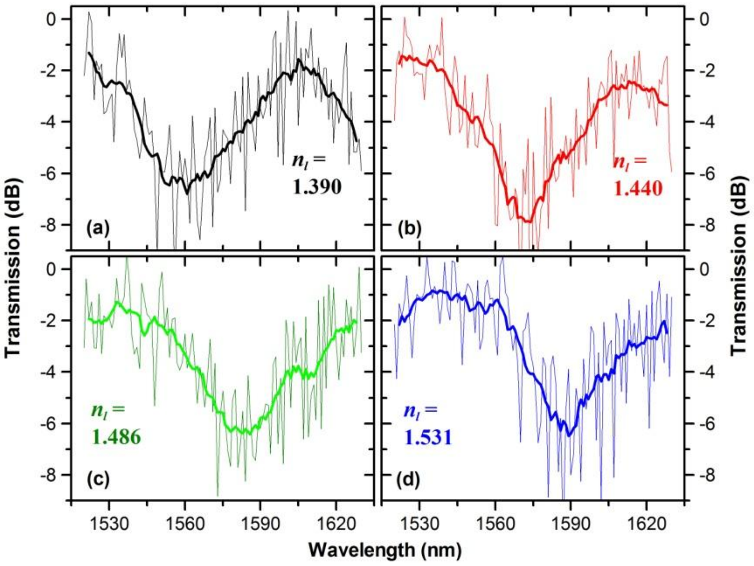

Figure S3. As-measured (thin lines) and smoothed (thick lines) transmission spectra for n_l = 1.390, 1.440, 1.486, and 1.531

S2. Theoretical transmission spectrum of a resonator-coupled waveguide

A generic resonator-coupled waveguide is schematically shown in Fig. S4. The resonance mode of the resonator has the propagation constant β_D and the attenuation coefficient α_D in the azimuthal direction. The coupling between the waveguide and the resonator has fractional loss which is given by $1-a_C^2$. The magnitudes of the straight-through and cross coupling coefficients of the coupling are denoted by τ and κ, respectively. It is assumed that a relation $\tau^2+\kappa^2=1$ still holds for the coupling. Then, the transmission (= $|E_4/E_1|^2$) of the waveguide, T is expressed by

$$T = a_C^2 \frac{(\tau - a_C e^{-2\pi\alpha_D r_D})^2 + 4\tau a_C e^{-2\pi\alpha_D r_D} \sin^2(\pi r_D \beta_D + \theta/2)}{(1-\tau a_C e^{-2\pi\alpha_D r_D})^2 + 4\tau a_C e^{-2\pi\alpha_D r_D} \sin^2(\pi r_D \beta_D + \theta/2)}, \qquad \text{(S2)}$$

where θ is the phase angle of the straight-through coupling coefficient. The theoretical transmission spectrum calculated by using Eq. (S2) was fitted to the smoothed transmission spectrum of the MISIM waveguide coupled to the PDR. Prior to the fitting process, first, the

isolated PDR with r_D = 0.9 μm was analyzed by using the bend mode solver of FIMMWAVE (Photon Design) based on the finite element method. This analysis provided the effective index N_D (= $\beta_D/(2\pi/\lambda_m)$, where λ_m is the resonance wavelength) and group index N_g of the resonance mode. In addition, α_D associated with the isolated PDR was also found. The values of these parameters are summarized in Table S1. The intrinsic Q factor of the isolated PDR was calculated by using the relation $Q_i = \pi N_g / (\alpha_D \lambda_m)$, and its values are shown in Table S1. The value for n_l = 1.440 is smaller than that in Fig. 2(a), which is obtained by using the FDTD method. The value of α_D obtained from the bend mode solver seems to become rather large due to small calculation domain dimensions which have to be limited for the PDR analysis. In the fitting process, while β_D was set to the value obtained from the analysis of the isolated PDR, the values of a_C, α_D, τ, and θ were determined. The values of these fitting parameters are summarized in Table S1. By using α_D from the fitting process, Q_i was calculated, and its values are shown in Table S1. The loaded Q factor Q_l of the PDR coupled to the MISIM waveguide, which is given by the ratio of λ_m to the linewidth of the spectrum, was extracted from the fitted transmission spectrum, and its values are also shown in Table S1. Figure S5 shows the smoothed and fitted transmission spectra for n_l = 1.390, 1.440, 1.486, and 1.531 when r_D = 0.9 μm.

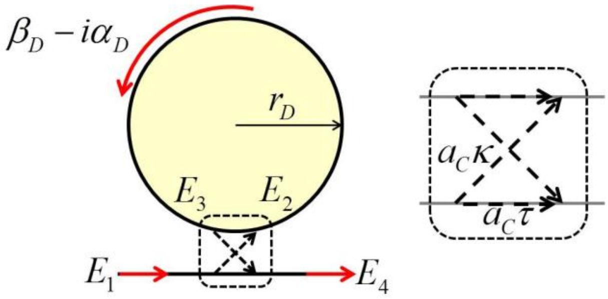

Figure S4. Schematic diagram of a generic resonator-coupled waveguide.

Table S1. Values of the fitting parameters

	n_l	1.390	1.440	1.486	1.531
Mode solver (FIMMWAVE)	N_D	2.033	2.060	2.088	2.114
	N_g	2.795	2.818	2.849	2.894
	α_D [μm^{-1}]	0.02578	0.02769	0.03062	0.03558
	Q_i	218	203	185	160
Fitting	a_C	0.8649	0.9299	0.9162	0.9738
	τ	0.8430	0.7888	0.8168	0.8216
	θ	4.06	3.79	3.46	3.07
	α_D [μm^{-1}]	0.07648	0.09908	0.102	0.1031
	Q_i	74	57	55	55
	Q_l	28.9	24.6	26.4	28.4

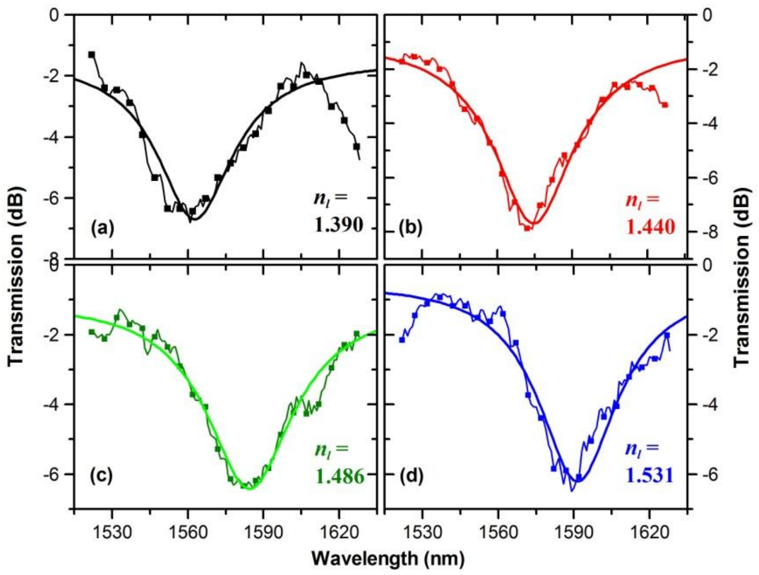

Figure S5. Smoothed (lines with symbols) and fitted (lines) transmission spectra for n_l = 1.390, 1.440, 1.486, and 1.531

S3. Dependence of the transmission spectrum on the air gap height *h*

The transmission spectra of the PDR-coupled MISIM waveguide with air gaps of height h at the bottom of the channels (See the inset of Fig. 5(d)) were calculated for a few values of h and n_l = 1.390, 1.440, 1.486, and 1.531. The calculated spectra are shown in Fig. S6. For a fixed value of n_l, the transmission spectrum blue-shifts as h increases. In addition, the minimum transmission at the resonance wavelength increases as h increases. For a larger value of n_l, the coupling between the MISIM waveguide and the PDR is more affected by the air gaps. Therefore, the decrease of the resonance wavelength becomes more significant for a larger value of n_l.

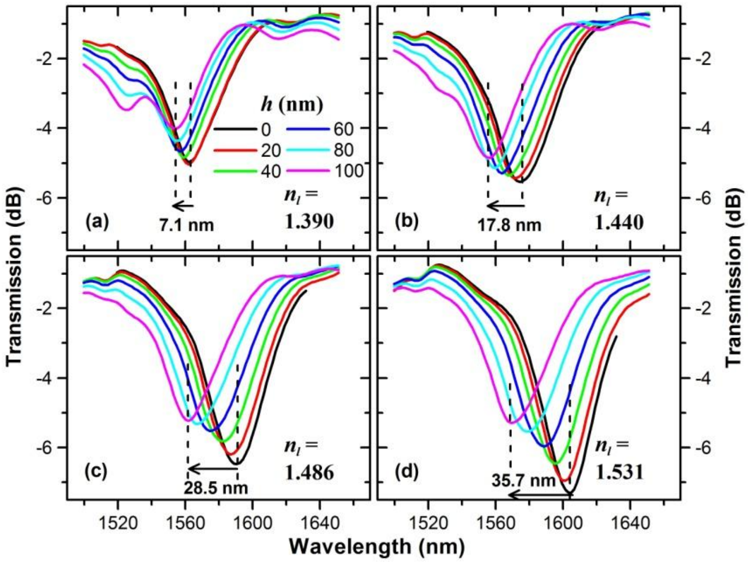

Figure S6. Transmission spectra of the PDR-coupled MISIM waveguide with air gaps of height h for a few values of h and n_l = 1.390, 1.440, 1.486, and 1.531. h varies from 0 to 100 nm in steps of 20 nm.

S4. Dependence of the transmission spectrum on w_a and l_a for w_S = 190 nm

The transmission spectrum of the MISIM waveguide with w_S = 190 nm which is coupled to the PDR with r_D = 0.9 μm was calculated for l_a = 0.23 μm and various values of w_a. In

addition, it was also calculated for w_a = 0.6 μm and various values of l_a. The calculated spectra are shown in Fig. S7. The transmission at the resonance wavelength does not change significantly depending on l_a. In contrast, it strongly depends on w_a. The transmission spectrum was calculated for various values of w_a and l_a other than those related to Fig. S6, but the transmission at the resonance wavelength was larger than –10 dB.

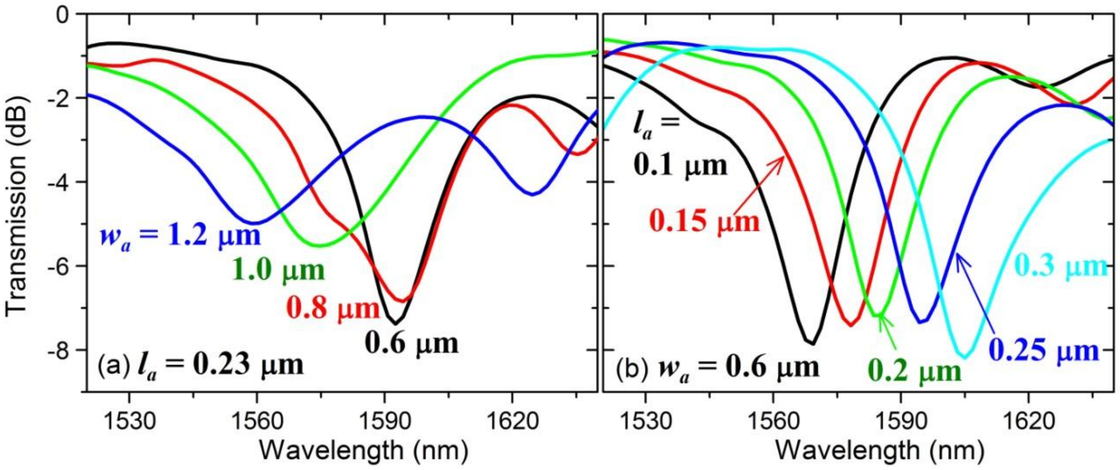

Figure S7. Transmission spectra of the MISIM waveguide with w_S = 190 nm which is coupled to the PDR with r_D = 0.9 μm for various values of (a) w_a and (b) l_a.

S5. Intensity modulator based on the PDR filled with liquid crystal

It may be challenging to fill the narrow channels with LC. However, if LC fills the channels, LC molecules are expected to align in the azimuthal direction[1-3]. By applying voltage between the metal and the Si disk (doped appropriately for electric conduction), the LC molecules are radially aligned. Therefore, the refractive index experienced by the major electric field component of the resonance mode (E_r) changes from the ordinary index n_o of the LC to the extraordinary index n_e. In the case of well-known LC E7, n_o and n_e are 1.5 and 1.68,

respectively[4]. The MISIM waveguide with w_S = 50 nm which is almost critically coupled to the PDR with r_D = 0.9 μm is considered here. Without the driving voltage, n_l is assumed to be equal to n_o. For n_l = 1.5, the transmission spectrum of the PDR-coupled MISIM waveguide was calculated. It is shown in Fig. S8. The resonance wavelength exists at 1582 nm. With the driving voltage, n_l is assumed to be equal to n_e. For n_l = 1.68, the transmission spectrum was also calculated. As shown in Fig. S8, the transmission at the resonance wavelength increases by 17.4 dB when n_l increases from 1.50 to 1.68. Consequently, the MISIM waveguide coupled to the LC-filled PDR can function as an intensity modulator with a large transmission change. Previously, it was shown that LC molecules filling a narrow channel are almost perfectly aligned perpendicular to the channel surfaces if the voltage across the channel surfaces is a few volts[1-3]. Therefore, the MISIM waveguide coupled to the LC-filled PDR may pave the way to an ultracompact intensity modulator operated with low driving voltage.

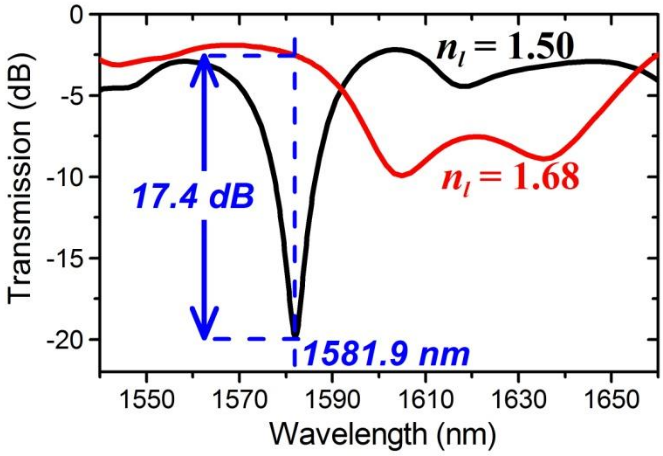

Figure S8. Transmission spectra of the MISIM waveguide with w_S = 50 nm which is almost critically coupled to the PDR with r_D = 0.9 μm for n_l = 1.5 and 1.68.

References

1. Pfeifle, J., Alloatti, L., Freude, W., Leuthold, J. & Koos, C. Silicon-organic hybrid phase shifter based on a slot waveguide with a liquid-crystal cladding. *Opt. Express* **20**, 15359-15376 (2012).
2. Zografopoulos D. C. & Beccherelli, R. Liquid-crystal-tunable metal–insulator–metal plasmonic waveguides and Bragg resonators. *J. Opt.* **15**, 055009 (2013).
3. Xing, Y. *et al*. Digitally controlled phase shifter using an SOI slot waveguide with liquid crystal infiltration. *IEEE Photon. Technol. Lett*. **27**, 1269-1272 (2015).
4. Cai, D.-P., Nien, S.-C., Chiu, H.-K., Chen, C.-C. & Chieh, C. Electrically tunable liquid crystal waveguide attenuators. *Opt. Express* **19**, 11890-11896 (2011).
